# Seeing the human behind the sample: How compassion training shaped inner awareness, relationships, and workplace meaning in HIV end-of-life research

**DOI:** 10.1177/26323524261467424

**Published:** 2026-07-08

**Authors:** Niamh Higgins, Whitney Tran, Rachel Lau, Joyce Lai, Jeff Taylor, Sara Schairer, Sara Gianella, Ali Ahmed, Karine Dubé

**Affiliations:** 1 Skaggs School of Pharmacy and Pharmaceutical Sciences, University of California San Diego (UCSD), La Jolla, CA, USA; 2 AntiViral Research Center (AVRC), 8784 UCSD, San Diego, CA, USA; 3 Center for Compassionate Communication, T. Denny Sanford Institute for Empathy and Compassion, UCSD, La Jolla, CA, USA; 4 Division of Infectious Diseases and Global Public Health, Department of Medicine, UCSD, La Jolla, CA, USA; 5 AVRC Community Advisory Board (CAB), UCSD, San Diego, CA, USA; 6 HIV+Aging Research Project-Palm Springs (HARP-PS); 7 Centers for Integrative Health, 657770 University of California San Diego Center for Mindfulness; 8 Compassion It, San Diego, CA, USA; 9 San Diego State University, San Diego, CA, USA

**Keywords:** compassion, empathy, burnout, self-compassion, end-of-life research, palliative care, compassion training, professional wellbeing, qualitative research, job satisfaction, Non-MeSH headings

## Abstract

**Background:**

Professionals engaged in HIV research at the end-of-life face ongoing emotional demands, yet there is limited evidence on whether brief compassion-based interventions can support resilience and team functioning in this context.

**Objective:**

To explore how members of the Last Gift team experienced a brief compassion training program and to examine its perceived value and acceptability within an emotionally demanding end-of-life HIV research setting.

**Methods:**

We conducted this qualitative study within the Last Gift, a rapid research autopsy program involving people with HIV and life-limiting illness at the University of California San Diego. Following completion of a four-week compassion training program, we held three focus group discussions with interdisciplinary team members (n = 10). We audio-recorded, transcribed, and analyzed discussions using conventional content analysis.

**Results:**

We identified three major themes: (1) intrapersonal growth and improved inner awareness when navigating emotionally intense work; (2) changes in interpersonal relationships with enhanced capacity to extend and receive compassion; and (3) strengthening community and shared purpose in workplace culture. Participants described applying compassion practices both at work and in daily life, contributing to improved interpersonal interactions, reduced emotional reactivity, and greater workplace satisfaction.

**Conclusions:**

Brief compassion training programs may offer a feasible and meaningful approach to supporting well-being, emotional resilience, and team cohesion among professionals working in end-of-life HIV research, particularly when embedded within supportive organizational contexts. Further research is needed to examine sustainability and broader implementation in palliative and end-of-life research settings.

## Plain Language Summary

Why was the study done? Professionals working in HIV research at the end of life regularly engage with participants who are seriously ill and dying. While this work is deeply meaningful, it can also be emotionally demanding. There is limited research on how to support the well-being and resilience of professionals in these settings. We wanted to understand whether a brief compassion training program could help. What did the researchers do? We offered a four-week compassion training program to members of the Last Gift research team, a rapid research autopsy program involving people with HIV and life-limiting illness at the University of California San Diego. After the program, we conducted group discussions with team members to learn about their experiences. We analyzed these discussions to identify common themes. What did the researchers find? Participants described greater self-awareness and improved ability to manage difficult emotions. They reported stronger relationships with colleagues, increased ability to give and receive compassion, and a deeper sense of shared purpose within the team. Many shared that they used the practices both at work and in their personal lives, leading to less emotional reactivity and greater workplace satisfaction. What do the findings mean? A brief compassion training program may be a practical and meaningful way to support emotional resilience and team connection among professionals working in end-of-life research. Supportive workplace cultures may further strengthen these benefits. More research is needed to examine long-term impact and broader use in palliative care and research settings.

## Key statements

### What is already known on this topic

• Professionals working in end-of-life healthcare and research settings experience high emotional demands and are at risk for burnout.
• Compassion- and mindfulness-based interventions have been shown to support emotional well-being and resilience among healthcare professionals, primarily in clinical care settings.

There is limited qualitative evidence describing how brief compassion training programs are experienced by interdisciplinary research teams engaged in end-of-life work.

### What this paper adds

• This study provides in-depth qualitative insight into how a brief, 4-week compassion training program was experienced by an interdisciplinary team working in an HIV rapid research autopsy program.
• Participants described changes in individual awareness and emotional regulation and in interpersonal relationships, team cohesion, and workplace culture.

### Implications for practice, theory or policy

• Brief compassion training programs may be a feasible and acceptable approach to supporting well-being among professionals working in emotionally demanding palliative and end-of-life research settings.
• Organizational context, such as leadership presence and shared purpose, is essential in determining how compassion practices are integrated and sustained.
• Institutions supporting end-of-life care or research should consider embedding compassion-based practices within organizational structures to promote resilience and sustainable work environments.

## Introduction

Occupational burnout is characterized by emotional exhaustion, depersonalization, reduced sense of personal efficacy, and is associated with demanding work environments, including heavy workload, limited recognition, job insecurity, and emotionally intensive work.^1–4^ Burnout can negatively affect personal wellbeing, workplace functioning, and workplace retention.^5^ Although the literature on burnout among research professionals is limited, evidence suggests that clinical research coordinators experience burnout at rates comparable to other healthcare workers.^2,6^ Research staff in intensive settings report psychological distress and occupational strain, with studies also identifying impaired work performance and intentions to leave the profession.^7,8^ Research personnel are often overlooked in wellbeing initiatives despite their essential role in clinical and translational science.

Mindfulness-based interventions are strongly correlated to psychological well-being, in part through enhanced self-compassion.^9^Self-compassion, defined as being supportive to oneself when experiencing difficult times,^10^ is strongly associated with greater happiness, optimism, and positive affect, and offers protection against acute stressors.^9^ Compassion cultivation training (CCT^TM^) is an 8-week interactive program developed at Stanford University^11^ which may improve self-reported mindfulness, self-compassion, compassion towards others, and interpersonal conflict in healthcare workers.^12^

The University of California San Diego (UCSD) Last Gift program is a distinctive end-of-life research cohort that partners with altruistic individuals with HIV who provide informed consent to rapid post-mortem tissue donation to advance knowledge on HIV cure-related research.^13,14^ The research and clinical teams operate under demanding logistical conditions, remaining on call to initiate a rapid research autopsy ideally within six hours of death, and often working extended shifts to complete complex procedures. Effective collaboration and mutual reliance are essential to carrying out this work.^13^ To date, the Last Gift research team has performed 46 rapid research autopsies since the advent of the program in 2017. The emotional demands of this work place team members at a heightened risk of empathic strain, psychological stress, and occupational burnout.

This study explores how a four-week, facilitator-guided compassion training program was experienced by members of the Last Gift team. The short intervention was designed to make compassion training more accessible. The purpose of the program was to cultivate compassion for oneself and others and to support professional well-being, with the broader goal of mitigating burnout and enhancing quality of life in this uniquely demanding research setting. A further aim was to generate preliminary insights into the acceptability and perceived value of a brief compassion-based program for individuals working in end-of-life HIV research.

## Methods

### Setting, participants recruitment and data collection

We employed a qualitative study design using focus group discussions (FGDs). Last Gift Study team members and collaborators were invited by email to participate in a compassion training program conducted during work hours. Inclusion criteria were: 1) being a Last Gift Study team member or collaborator, 2) ability to read English, 3) provision of informed consent. Exclusion criteria included any individuals experiencing recent traumatic life circumstances that could cause daily meditation practice to be overwhelming. Participation was voluntary and did not affect employment. Supervisors did not attend the sessions to minimize perceived pressure, coercion, or hierarchical bias.

Following informed consent, willing participants enrolled in a four-week compassion training program led by a facilitator (S.S.) from the UCSD Center for Mindfulness. The program consisted of four 90-minute sessions delivered weekly via Zoom and covered mindfulness, self-compassion, common humanity, and active compassion. The facilitator adapted the Compassion Cultivation Training (CCT™) curriculum to a condensed four-week format to improve feasibility and accessibility for participants while retaining core instructional components. Selected content from self-compassion, compassion for others, and integrated daily compassion practice modules was combined across sessions to preserve key compassion-focused principles and practices.

The intervention was informed by a conceptual theory of change in which mindfulness and compassion practices were expected to help participants become more aware of their own emotions, respond to themselves with greater kindness, and recognize shared human experiences. These changes were expected to support emotional regulation, reduce self-criticism, increase openness to receiving support, and help participants respond more compassionately to colleagues and others. At the team level, the intervention was expected to strengthen communication, connection, shared purpose, and workplace resilience in the emotionally demanding context of end-of-life HIV research.

In addition to facilitator-led sessions, participants were assigned daily guided meditation practices of approximately 15 minutes and were encouraged to integrate informal mindfulness and compassion practices into daily activities.

Upon completion of the compassion training program, all participants were invited to take part in FGDs to share perspectives on their experiences. As a qualitative study, no formal statistical power calculation was performed. Participants were recruited from among individuals who completed the compassion training and were willing to participate in post-intervention FGDs. The recruitment stopping point was determined by participant availability and completion of three FGDs, rather than by prospective use of thematic saturation as a stopping criterion. Among 24 participants who participated in the compassion training, 10 participated in one of three FGDs between December 2024 and June 2025. FGDs were conducted a median of 8 weeks following completion of the training program (range: 3–12 weeks).

### Focus group discussion guide

Members of the research team (N.H. and K.D.) developed the FGD guide by drawing from a question bank derived from previously published qualitative studies.^13^ The question bank was not a previously validated questionnaire; rather, it was adapted for this study context based on prior qualitative literature and expert review by the research team. We reviewed each item for relevance to the context of HIV research at the end of life and compassion training, modified wording as needed, and added new items to address themes specific to the intervention. The full discussion guide is provided in Supplementary Table 1.

## Data collection

Two female facilitators (N.H. and K.D.) and one male facilitator (J.T.) with prior experience in qualitative research and complementary disciplinary backgrounds in clinical pharmacy, socio-behavioral science and community advocacy conducted three FGDs. Facilitators were professional colleagues of participants through the Last Gift research program but were not in supervisory roles, and participation was voluntary and unrelated to performance evaluation. Participants were informed that the purpose of the FGDs was to explore their perceptions and experiences of the Last Gift Compassion Training program.

With only facilitators and participants present, the FGDs were conducted in English using the Zoom videoconferencing platform and they lasted approximately 60–90 minutes. We audio-recorded sessions with participant permission. We did not collect protected health information. During each FGD, one facilitator led the discussion while the other took detailed field notes and assisted with logistics.

### Data analysis

Our team stored audio recordings on a secure, access-restricted server and transcribed using an encrypted transcription service. We did not return transcripts to participants for comment; trained research assistants (W.T., R.L., J.L.) reviewed transcripts for accuracy by comparing them with the original recordings and removed any identifying information.

We used conventional content analysis^15^ as the guiding qualitative analytic framework because the study was exploratory and sought to derive codes, categories, and themes inductively from participant narratives rather than apply a pre-existing theoretical framework. To strengthen reporting transparency, we reviewed the manuscript using relevant items from the Consolidated Criteria for Reporting Qualitative Research (COREQ) Supplementary Table 2.^16^ Multiple members of the research team (A.A., W.T., J.L., N.H.) independently coded transcripts without applying a pre-existing coding framework due to the exploratory nature of the study. Research assistants (W.T., J.L.) and the principal investigator (N.H.) developed an initial codebook, which was reviewed and refined by a secondary coder (A.A.). Initial codes were iteratively reviewed, grouped into broader categories, and synthesized into overarching themes. Our team discussed coding discrepancies during bi-weekly meetings (N.H., A.A., K.D.) and resolved them through consensus. We also reviewed less common or divergent accounts during coding and consensus meetings. No deviant themes emerged that contradicted the central interpretation of the intervention as feasible, acceptable, and meaningful to participants. However, variation in participants’ experiences was retained in the analysis and considered during theme development. Throughout the analytic process, the research team engaged in reflexive discussions to acknowledge how prior involvement with the Last Gift study and the principal investigator’s prior experience with compassion-focused work could influence interpretation of participant narratives. Use of multiple coders and consensus discussions helped enhance analytic rigor and minimize the influence of individual perspectives on data interpretation. During analysis, major themes recurred across FGDs, with no substantially new major themes emerging in later discussions. Formal member checking was not performed because of the exploratory nature of the study and the small, workplace-based participant group. Instead, trustworthiness was supported through transcript review for accuracy, independent coding by multiple team members, iterative codebook refinement, reflexive discussions, team-based review of emerging themes, and consensus-based resolution of discrepancies.

We present representative quotes in the Results section, with additional supporting quotes provided in Supplementary Table 3.

### Ethical considerations

The UC San Diego Office of Institutional Review Board Administration reviewed the Last Gift Compassion Training protocols (#810327 and #811193) and determined that the study met criteria for exempt status.

## Results

Ten participants from the intervention participated in one of three focus groups. Most were female at birth (8/10) and between the ages of 25 and 44 years (7/10) (Table 1). The sample was ethnically diverse (3/10 Asian, 2/10 Caucasian, 2/10 Hispanic descent; 2/10 Multiracial/Other, 1/10 declined to disclose) and reported having roles (non-mutually exclusive) that were clinical (4/10), laboratory-based (5/10), administrative (2/10) and research-based (4/10). Six participants reported attending rapid research autopsies. Seven had previous meditation experience and three reported having a routine seated meditation practice.

**Table 1.** Demographic characteristics of study participants. Median (IQR) shown for continuous variables and count (percentage) for categorical variables.

Characteristic	N = 10
**Age at Enrollment (Years)**	
25 to 34	3
35 to 44	4
45 to 54	3
**Sex at Birth**	
Male	2
Female	8
**Race/Ethnicity**	
Asian	3
White/Caucasian	2
Other or Multiracial	2
Hispanic, Latino or Spanish Origin	2
Declines to State	1
**Education**	
Some College, no Degree	1
Bachelor’s Degree	3
Master’s Degree	1
Doctorate or Professional Degree (e.g. MD, PharmD)	5
**Work Setting**	
Clinical	
Yes	4
No	6
Laboratory	
Yes	5
No	5
Research	
Yes	4
No	6
Administrative	
Yes	2
No	8
Attends Rapid Research Autopsies	
Yes	6
No	4
Previous Meditation Experience	
Yes	7
No	3
Routine Seated Meditation Practice	
Yes	3
No	7

Our data revealed three major themes: 1) intrapersonal growth and inner awareness, with subthemes related to enhanced mindfulness/self-compassion and receiving compassion, 2) interpersonal and community connections, with subthemes relating to extending compassion, recognizing common humanity and navigating interpersonal conflicts, 3) the application of compassion skills to the workplace and everyday life, with subthemes related to community/team dynamics and workplace stress/satisfaction.

### 1. Intrapersonal growth and inner awareness

FGDs reflected pronounced internal changes resulting from the compassion training. Participants reported deeper engagement in mindfulness, stronger self-compassion, and broadened ability to respond to compassion.

#### Cultivating mindfulness

Participants described how regularly engaging in brief moments of mindfulness produced noticeable benefits for professional and personal well-being. They discussed a strengthened ability to direct focus into their internal state, grounding themselves in more present-moment and bodily awareness.

*I would say the benefits I found were that I was able to turn the focus inward a bit, which initially felt a bit selfish and yes, and at the same time, it made me realize that I need to do that to positively impact myself and the others around me. And so, whether it's personal or professional… I'm able to actually, like somatically, drop into my body and listen to my body in a compassionate way and make a decision without spinning in my brain as much as I did before*. – LG-CT-FGD-16

Participants also described these internal shifts as playing a central role in cultivating compassion, for oneself and others. Participants noted increased reflective awareness and intentional presence in response to emotionally demanding aspects of their work. One participant described how this reflective stance shaped their choices around participation in rapid research autopsies, reframing presence as a compassionate act that honored the participants’ final gift:

*[W]hen this happens, I feel like I am very reflective, and I think to myself… one person is dying today… and I have the option to honor their body and their gift by being present… being present in the autopsy is honoring their gift… it changes my perspective on… what this gift means… and it always ends up making me want to go… to honor them, right?*— LG-CT-FGD-07

#### Cultivating self-compassion

Participants reported increased self-compassion, describing an enhanced ability to focus inward, address their own needs without guilt, and recognize their own accomplishments. Some remarked on how the compassion training mitigated negative internal language and feelings of selfishness, helping to maintain boundaries between personal and work-related demands. Reducing negative self-evaluation also opened participants to receiving compassion from others.

*I think for a long time, I was putting a lot of pressure on myself … I don't know why. And that was making me very sick, very like physically sick. And I think that through this training, I was able to give myself grace and just kind of like, yeah, just be compassionate towards myself… and recognize how much I've already accomplished… And that helped me heal, not only like physically, but like mentally. And so it's… a healing journey to be compassionate towards yourself. —* LG-CT-FGD-07

#### Becoming more open to accepting/responding to compassion

Participants reported noticeable improvements in their ability to accept and respond to compassion from others. Common narratives described shifts in perceptions of oneself as burdensome, subject to others’ pity, and vulnerable to exploitation to the worthiness of others’ compassion.

To navigate feelings of being a burden, participants reflected on the motives underlying their compassion toward others. A recurring strategy involved substituting apologies with expressions of gratitude, which helped to normalize receiving support from others.

*Somebody had mentioned you don't want to be a burden to them. You don't want them to necessarily have to worry about you. But I think again, we're deserving of the compassion that we give to others… slowly being more comfortable with accepting whether it's praise or something like that.*— LG-CT-FGD-01

Additionally, participants explored concerns about social evaluation, including anticipated assessments of weakness by others, when addressing discomfort with unwanted attention and being perceived as pitiful. Another respondent challenged prior assumptions of others’ compassionate gestures as self-serving.

*I think prior to the training, I almost felt like people were taking pity on me, or people thought I was weak if I needed compassion… and granted, four weeks is not a long time to create a complete mindset shift, but there's a bit of a drift in the mindset where you know, receiving compassion doesn't make you weak… people won't perceive you to necessarily be weak… and people aren't going to take advantage of it. —* LG-CT-FGD-25

Overall, the compassion training supported participants through deconstructing negative preconceptions of receiving compassion and fostering greater openness to accepting and responding to support from others.

### 2. Interpersonal and community connections

Participants also reflected on noticeable shifts in navigating interpersonal dynamics. Focus group participants described heightened awareness of common humanity, enhanced ability to extend compassion to others, and improved interpersonal conflict management.

#### Recognizing common humanity & extending compassion to others

Participants described how shared reflections during the compassion training enhanced feelings of common humanity. They reflected on adversity as a fundamental pillar of the human experience and became more aware of the less visible struggles carried by all. Through mindfulness exercises, participants helped to mitigate negative feelings associated with perceived personal shortcomings and comparison to individuals whose difficulties were not readily observable. As participants reported an increased awareness of common humanity, they also described shifts in their capacity to extend compassion to others.

*To hear them express some of their vulnerabilities and thoughts and fears did… reinforce that we really… do have some shared human experience and shared humanity, and a lot of us are going through the same things, no matter what face we put forward… it also helped me to share more compassion to others, recognizing that… my struggles are not just… a personal failing, and that if I'm able to share that and extend some grace and anticipate where others may have similar doubts or fears, by being able to reach out, I think that's helped me support others as well.*— LG-CT-FGD-09

Discussions about the unseen challenges of others highlighted small acts of compassion, with participants indicating that helping others feel seen was central to how they practiced compassion toward strangers.

There's sort of this assumption that everybody's going through something you just don't know what, whether they're being difficult or not, even just at the grocery store, someone ringing you up. I just try to ask how their day is going, or something like that *—* LG-CT-FGD-01

For participants who described their relations with loved ones as difficult, compassion was enacted by creating space for listening and common understanding.

*I still love them, they still love me, and still connecting to what we do have in common… and trying to see where they're coming from, and understanding how much fear they have, and kind of, instead of the immediate reaction of all of my emotions, I'm able to step back a little bit and see a little bit more of where they're coming from for myself. —* LG-CT-FGD-09

Some also expressed a desire to demonstrate compassion to loved ones through example and active compassion. Overall, responses indicated that the compassion training improved participants’ ability to recognize common humanity and extend compassion across unfamiliar, close, and challenging relationships.

#### Navigating interpersonal conflicts

A prominent thread across FGDs was the impact that the compassion training program had on navigating interpersonal challenges. Participants described an improved capacity to respond compassionately to challenging interactions by reframing others’ behavior as unrelated to themselves, which supported greater equanimity in managing difficult relationships.

*I think once you cannot take anything personal, you unlock, like, this whole level of, like, peace. If you think to yourself that maybe this person is going or struggling through something we know nothing about. And even more so maybe nobody's ever shown them compassion. And so rather than again, making it about you, just it's not about me, they're going through something. I feel like they need probably more compassion than anybody else at that moment for sure, that's my approach.*— LG-CT-FGD-01

Participants’ responses described managing conflict with more patience, emotional regulation, and open curiosity. Some described pausing before responding and softening emotional intensity within difficult interactions.

*I think the change that I've noticed… is that I'm leading with less… extremes of emotional ends of the spectrum. I feel like my emotions are kind of living this like 30-degree arc in the middle now, when I have interpersonal conflict, and now I'm able to, kind of like, slow it down, take a couple deep breaths, do the… one exercise, “just like me”. —* LG-CT-FGD-25

Practicing compassion across diverse interpersonal relationships also translated to workplace culture, as participants described increased confidence in navigating challenging interactions, contributing to more harmonious team dynamics.

*One thing this study showed me is that… it empowered me… I can change that relationship with the difficult people. And there's another word, it gave me the courage, right? —* LG-CT-FGD-15

### 3. Applying compassion skills to work and everyday life

Participants noted feeling a stronger sense of community and team functioning among Last Gift colleagues. They described these relational changes as being both individually supportive and beneficial to the entire team. This strengthened sense of collective effort was associated with increased workplace satisfaction and diminished stress among participants.

#### Building community in workplace culture

A deeper sense of community within the Last Gift team was a noteworthy subtheme. The compassion training program was consistently described as a setting where vulnerability was permissible and shared emotional experiences helped normalize the inherent work challenges. Accountability buddies further reinforced bonds and sustained engagement with the home practices.

*I think that mostly it's that sense of community and kind of shared vulnerability and closeness that developed… we were all vulnerable, and we trust that… what was said in the training… I don't see any of the participants for the compassion training, violating trust*— LG-CT-FGD-09

Participants also reported meaningful shifts in everyday team interactions, such as increased honesty, stronger emotional attunement, and improved communication. Some participants spoke about checking in with colleagues more frequently, and offering support during stressful moments, especially during the rapid research autopsies. Participants noted more ease in emotional transparency and a willingness to voice personal needs.

*I think it made being honest a lot [easier] between us… I feel like before it was a little more difficult to tell each other that we needed a break. And I think after this, it's a lot easier to tell one another… “Hey, I need a break”*— LG-CT-FGD-02

These changes in team dynamics were perceived to enhance workplace efficiency and reduce interpersonal barriers.

### Supporting stress management, resilience & workplace satisfaction

Participants reported no change or a reduction in stress following the compassion training. Normalizing challenges and fostering mutual understanding among colleagues appeared to mitigate stress by easing feelings of isolation at work.

Additionally, discussion recurrently highlighted how the training equipped participants with self-compassion strategies to set boundaries between work demands and personal life. Several mentioned improved abilities to prioritize personal needs without guilt:

*Before I'd be like… really frustrated, and, you know, anxious about leaving a little bit early to meet friends on a busy clinic day, but I'm like, “You know what? No, this is what I need to do for me, and that's okay.” I can tell myself that I will get it done, and that's okay… It's a lot more compassion for myself and what I need versus what the external needs to take from me. It's like I need to fill my cup first in order to give my best to everyone, to everything and everyone else, and without feeling guilty about it, and then allowing myself to be present when I am in the presence of people that I care about as well.*— LG-CT-FGD-16

Moreover, focus group participants reported increased pleasure from performing their job well. Participants described reconnecting to the humanistic and participant-centered dimensions of the Last Gift program, deepening a feeling of purpose in daily tasks.

*It helps me… see the sample as… somebody that was a human… it makes me see it with some more intent, even with… emotional purpose. It helps me to have some sort of, like dedication to the sample and make sure that… I'm doing my work with integrity so that the best results are produced and we can contribute the most… to like find a cure to HIV. —* LG-CT-FGD-02

Connection with the broader mission of their work was reinforced as the compassion training exposed participants to colleagues outside their immediate teams. Recognizing the collective effort and shared purpose behind the Last Gift program helped participants derive renewed energy and meaning from their work, buffering against burnout and enhancing overall workplace satisfaction.

## Discussion

### Main findings

Given the emotional intensity and ethical complexity of end-of-life research, there is a need to better understand how brief compassion-based interventions may support the well-being and functioning of research professionals. Participants shared rich narrative data which suggest that even short compassion programs can still have meaningful impact, linking practices to personal wellbeing and team functioning. Participants described the impact of the compassion training program on personal growth and inner awareness. Responses reflect deepened engagement in mindfulness, improved self-compassion and emotional resilience, and greater openness to accept compassion from others. These emergent outcomes facilitated emotional regulation in interpersonal dynamics and stress resilience at work. Participants discussed the importance of formal and informal practices. They described how turning inward allowed for reflective awareness and a renewed commitment to the Last Gift research mission. Enhanced self-compassion helped to create healthy boundaries, to improve work-life balance, and to strengthen emotional resilience. In addition, the ability to more freely accept compassion, as noted by our participants, could allow one’s distress to become shared and normalized. This process directly counters isolation and over-identification (i.e., the tendency to get carried away by negative emotions), two elements that are linked to burnout and emotional exhaustion.^17^ Thus, compassion-based interventions can change one’s relationship with difficult emotions and stress, which invariably affects how one relates to others.

Our findings further demonstrate that compassion training can create changes in interpersonal relationships and ignite shared purpose, functioning as a social intervention in the workplace, rather than simply an individual one. Participants described a stronger sense of community, enhanced communication, and an improved ability to manage interpersonal conflict. The meaningful changes that participants experienced also appeared to strengthen collaboration, workplace efficiency (e.g., during rapid research autopsies), in addition to well-being. Discussion responses also conveyed positive team dynamics, which contributed to greater workplace satisfaction and stress resilience. In a systematic review of healthcare professionals, compassion-related interventions showed improvements in interpersonal conflict scores.^18^ Other studies have shown that shared experiences and compassion practices can strengthen interpersonal relationships and create a sense of community.^19,20^ Responses further reflect the relevance of collective purpose as a means of mitigating personal and professional burnout. Notably, mission-focused intentionality has been described as a key element in decreasing burnout and increasing compassion satisfaction in emotionally intense work environments.^21^

While our results align with a growing body of evidence that brief compassion and mindfulness-based interventions can support professionals working in emotionally intense settings^22,23^ they further extend the compassion-training literature to end-of-life research contexts. Our qualitative findings complement and expand results from our previously published quantitative evaluation of the Last Gift Compassion Training program, which identified improvements in overidentification, workplace joy, and the perception of a supportive work environment.^24^ While the quantitative data detected measurable changes over 12 weeks, the current qualitative data offer insight into *how* participants experienced these changes, including shifts in emotional awareness, interpersonal interactions and approaches to emotionally demanding work. Emerging themes from the FGDs shed light on how participants integrated compassion practices within, and balancing between, their professional and personal lives.

Beyond individual coping strategies, organizational context can play a role in reducing staff burnout. One retrospective study explored antecedents (e.g., workload, fairness, reward, community) of burnout in two clinical trial call centers.^25^ Lower turnover and higher consent rates were observed when staff received fair compensation, experienced less pressure to make a certain number of calls, were given a greater variety of responsibilities, and participated in project decisions. Among oncology professionals, leadership endorsement and active facilitation were essential to participation in compassion training.^22^ In a national study of clinical research professionals working in mental health, the perception of a supportive organizational climate, particularly one that prioritized research ethics and team support, buffered the effects of moral stress and burnout. Similarly, the Last Gift study presents ethical commitments at the end of life and emotional demands that may extend beyond staff roles and can deeply affect research professionals. The Last Gift program has long prioritized team well-being through supportive leadership practices, including positive reinforcement, fair compensation, community engagement, and cultivating a shared sense of purpose through direct connection with participants and their families. This positive organizational climate may have amplified the impact of the compassion training by reinforcing mutual respect, recognition, and collective meaning in an emotionally demanding work environment. Thus, compassion-based interventions must be paired with a broader institutional commitment to staff wellbeing.

#### Limitations

This study has several limitations. First, the small sample size limits generalizability. The FGD component aimed to explore experiences with the compassion training program rather than produce statistically generalizable findings. Although the discussion guide was informed by prior qualitative literature and reviewed by experienced investigators, it was not formally pilot-tested prior to use. Self-selection into the program could reflect participants who were already motivated toward compassion practices. Moreover, although 24 team members participated in the Last Gift Compassion Training program, only 10 opted to participate in FGDs, introducing the potential for participation bias. Participants also completed FGDs at varying time points following the intervention (3–12 weeks), which may have influenced the immediacy of reflections and reported experiences. Prior experience with meditation or related contemplative practices may also have influenced how participants engaged with and perceived the intervention. In addition, the intervention represented a condensed adaptation of the standard CCT™ curriculum, and findings may not directly reflect experiences associated with the full program. Finally, the study was conducted within a single research setting with a unique mission and team structure, which may limit transferability to other healthcare or research environments. Longer-term effects remain unknown and there are inherent limitations to the FGD model, including conformity bias.

### Considerations for implementation

These findings informed a set of practical recommendations for teams working in emotionally demanding settings (Table 2). Although our results reflect experiences during a 4-week intervention, participants described the mechanisms through which this training improved mindfulness, self-compassion, and team dynamics. Thus, there are potential applications that extend beyond the study timeframe. These examples are informed by the Last Gift Compassion Training program; participant experiences could vary and should be adapted to meet the needs of other teams.

**Table 2.** Insights from the Last Gift Compassion Training and practical applications.

Themes	Key Insights	Practical Applications	Implementation Considerations
Intrapersonal Growth and Inner Awareness	Participants described how practicing mindfulness formally and informally encouraged intentional presence at work.	Teams may consider offering brief, mindful moments into existing workflow (e.g. minute of silence, intention setting at the start of week).	Participation should be voluntary; benefit beyond 4 weeks was not assessed in this study.
Interpersonal and Community Connections	Recognizing common humanity helped participants to navigate challenging interactions.	Teams may offer skill-based workshops on compassion which could include teaching members about common humanity. Guided meditation and informal exercises (e.g. “just like me”) could be used to strengthen one’s ability to give compassion to others.	Ensuring psychological safety is critical.
Applying Compassion Skills to Work and Everyday Life	Opportunities for shared vulnerability strengthened a sense of community among team members.	Teams may consider creating structured opportunities for voluntary sharing; normalizing reflection; using team building/connection activities focused on shared purpose (the ‘why’ the work matters).	Explore ideas for sustained engagement (e.g. accountability partners, monthly meet-ups).
Interpersonal and Community Connections	Participants described feeling safer acknowledging emotional experiences when supported by shared practices and peer connection.	Examples include accountability buddies, drop-in reflection sessions, inviting reflection during meetings (e.g. one-word check-ins, mood check-ins), structured, facilitated debriefs during emotionally intense events (e.g. deaths, rapid research autopsies).	Accountability buddies should be self-selected.
			Leadership should model behavior.
			During reflections and debriefs, group facilitators should prioritize listening versus problem solving (e.g. no fixing).

## Conclusions

We found how a brief compassion training program supported emotional resilience, interpersonal relationships, and connectedness while working in an emotionally intense research environment. Notably, participants emphasized cultivating compassion for themselves and others but also developing the capacity to *receive* compassion from others—allowing one’s struggles to be validated and normalized, which in turn can reduce stress. Together with the participant-informed recommendations outlined in Table 2, these findings offer practical guidance for organizations seeking to support team wellness, communication, and sustainability in emotionally demanding research settings.

## Supplemental material

Supplemental material - Seeing the human behind the sample: How compassion training shaped inner awareness, relationships, and workplace meaning in HIV end-of-life research Supplemental material for Seeing the human behind the sample: How compassion training shaped inner awareness, relationships, and workplace meaning in HIV end-of-life research by Niamh Higgins, Whitney Tran, Rachel Lau, Joyce Lai, Jeff Taylor, Sara Schairer, Sara Gianella, Ali Ahmed, Karine Dubé in Palliative Care and Social Practice

Supplemental material - Seeing the human behind the sample: How compassion training shaped inner awareness, relationships, and workplace meaning in HIV end-of-life research Supplemental material for Seeing the human behind the sample: How compassion training shaped inner awareness, relationships, and workplace meaning in HIV end-of-life research by Niamh Higgins, Whitney Tran, Rachel Lau, Joyce Lai, Jeff Taylor, Sara Schairer, Sara Gianella, Ali Ahmed, Karine Dubé in Palliative Care and Social Practice

Supplemental material - Seeing the human behind the sample: How compassion training shaped inner awareness, relationships, and workplace meaning in HIV end-of-life research Supplemental material for Seeing the human behind the sample: How compassion training shaped inner awareness, relationships, and workplace meaning in HIV end-of-life research by Niamh Higgins, Whitney Tran, Rachel Lau, Joyce Lai, Jeff Taylor, Sara Schairer, Sara Gianella, Ali Ahmed, Karine Dubé in Palliative Care and Social Practice

## Data Availability

Due to participant confidentiality considerations and the conditions of informed consent, the qualitative data generated during this study are not publicly available. Requests for data will be considered on a case-by-case basis in accordance with institutional policies and ethical requirements.*
