# Supplementary material for: Seeing the human behind the sample: How compassion training shaped inner awareness, relationships, and workplace meaning in HIV end-of-life research
Source: Palliat Care Soc Pract. 2026 Jul 8;20:26323524261467424. doi: 10.1177/26323524261467424 (PMC13351235; doi:10.1177/26323524261467424)
Supplement: Supplemental material - Seeing the human behind the sample: How compassion training shaped inner awareness, relationships, and workplace meaning in HIV end-of-life research [file sj-pdf-1-pcr-10.1177_26323524261467424.pdf]

Supplementary Table 1: Focus Group Discussion Guide for Team Members of Last Gift Program who Participated in the Compassion Training Program

Introductory/General Questions

- First, thank you so much for your time.
- What did the Last Gift Compassion Training Study mean to you?

Focus Group Discussion Questions for Staff Involved in the Last Gift Compassion Training Study

- What does it mean to you to be part of the study as a staff member?
- What are your feelings about the LG-CT study?
- Has the LG-CT study changed how you conduct your research? If yes, how so?
- Did you develop/enhance a bond with the other participants in the LG-CT study? Can you please explain?
- Is there anything you wish you had known before your involvement with the LG-CT study? If so, what is it?
- Do/did you see any benefits/positives to the LG-CT participants of being in the study?
- Do/did you see any risks/negatives to the LG-CT participants of being in the study?

Compassion Training Outcomes

- Since completing the LG-CT study, did you feel any change in your ability to offer compassion to the following groups (please describe any changes):
    - Loved ones?
    - Oneself?
    - To difficult people?
    - To strangers?
- Can you comment on your home practice during the LG-CT study? Did you experience challenges to find time to commit to your home practice?
- Since completing the LG-CT study have you noticed any improvement in your ability to bring mindful awareness into your everyday activities? Please describe.
- Since completing the LG-CT study have you noticed any improvement in your ability to bring mindful awareness into your research activities with the Last Gift? Please describe.
- Since completing the LG-CT study have you noticed any changes in your willingness to accept/respond to compassion from other people?
- Since completing the LG-CT study, have you noticed any improvement in your approach to handling interpersonal conflicts? Please explain.
- Other people may share similar feelings when encountering difficult situations. Since completing the LG-CT study, have you noticed any shift in your perspective on the shared human experience? Please explain.

Professional Quality of Life and Burnout

- Since completing the LG-CT study have you noticed any changes in the pleasure you derive from being able to do your job well? Please describe.
- Since completing the LG-CT study have you noticed any changes in the degree to which the Last Gift team works efficiently together? Please explain.

Resilience/Coping

- How did the LG-CT study affect your stress? How did you deal with this stress?
- Did any aspect of participating in the LG-CT study create emotional distress? If so, please describe. How did you deal with this?
- Do you experience stress associated with your involvement in the Last Gift study? Did participation in the LG-CT study help you to cope with this stress?

Feasibility of Expanding the LG-CT

- How realistic do you believe it would be to implement a compassion training program that would involve Last Gift study participants? What about the caregivers of Last Gift study participants?
- Do you have any ideas or suggestions about developing a compassion training program that would accommodate the needs of the Last Gift study participants? What about the caregivers of the Last Gift study participants?

Ethical Considerations

- Do you see any ethical issues with staff members participating in the LG-CT study?
- Do you see any ethical issues with LG study participants or their caregivers participating in the LG-CT study?
- Did you have feelings of vulnerability during the LG-CT study? Please explain.

Ending Questions

- Do you have any recommendations to improve the conduct of the study?
- Can you think of anything else you would like to share with the group on this topic?

LG-CT: Last Gift Compassion Training
