# Supplementary material for: Seeing the human behind the sample: How compassion training shaped inner awareness, relationships, and workplace meaning in HIV end-of-life research
Source: Palliat Care Soc Pract. 2026 Jul 8;20:26323524261467424. doi: 10.1177/26323524261467424 (PMC13351235; doi:10.1177/26323524261467424)
Supplement: Supplemental material - Seeing the human behind the sample: How compassion training shaped inner awareness, relationships, and workplace meaning in HIV end-of-life research [file sj-pdf-2-pcr-10.1177_26323524261467424.pdf]

Supplementary Table 2: COREQ checklist

Consolidated criteria for reporting qualitative studies (COREQ): 32-item checklist

Developed from: Tong A, Sainsbury P, Craig J. Consolidated criteria for reporting qualitative research (COREQ): a 32-item checklist for interviews and focus groups. International Journal for Quality in Health Care. 2007;19(6):349-357.

Item No and topic	Guide questions/description	Reported in manuscript section / location
**Domain 1: Research team and reflexivity**		
**Personal characteristics**		
1. Interviewer/facilitator	Which author/s conducted the interview or focus group?	Methods > Data Collection, paragraph 1.
2. Credentials	What were the researcher's credentials? E.g., PhD, MD	Methods > Data Collection, paragraph 1 (disciplinary backgrounds); Author list and affiliations. Specific credentials/degrees for all facilitators are not fully reported.
3. Occupation	What was their occupation at the time of the study?	Methods > Data Collection, paragraph 1 (disciplinary backgrounds and roles); Author list and affiliations.
4. Gender	Was the researcher male or female?	Methods > Data Collection, paragraph 1.
5. Experience and training	What experience or training did the researcher have?	Methods > Data Collection, paragraph 1; Methods > Setting, Participants Recruitment and Data Collection, paragraph 2.
**Relationship with participants**		
6. Relationship established	Was a relationship established prior to study commencement?	Methods > Data Collection, paragraph 1.
7. Participant knowledge of the interviewer	What did the participants know about the researcher? e.g., personal goals, reasons for doing the research?	Methods > Data Collection, paragraph 1 (participants were informed of the purpose of FGDs). Further details on what participants knew about facilitators are not fully reported.
8. Interviewer characteristics	What characteristics were reported about the interviewer/facilitator? e.g., bias, assumptions, reasons and interests in the research topic	Methods > Data Collection, paragraph 1; Methods > Data Analysis, paragraph 2.
**Domain 2: Study design**		
**Theoretical framework**		
9. Methodological	What methodological orientation was stated to underpin	Methods > Data Analysis, paragraph 1.

Item No and topic	Guide questions/description	Reported in manuscript section / location
orientation and theory	the study? e.g., grounded theory, discourse analysis, ethnography, phenomenology, content analysis	
**Participant selection**		
10. Sampling	How were participants selected? e.g., purposive, convenience, consecutive, snowball	Methods > Setting, Participants Recruitment and Data Collection, paragraph 3.
11. Method of approach	How were participants approached? e.g., face-to-face, telephone, mail, email	Methods > Setting, Participants Recruitment and Data Collection, paragraph 1.
12. Sample size	How many participants were in the study?	Methods > Setting, Participants Recruitment and Data Collection, paragraph 3; Results, opening paragraph.
13. Non-participation	How many people refused to participate or dropped out? Reasons?	Methods > Setting, Participants Recruitment and Data Collection, paragraph 3. Reasons for non-participation are not reported.
14. Setting of data collection	Where was the data collected? e.g., home, clinic, workplace	Methods > Data Collection, paragraph 2.
15. Presence of nonparticipants	Was anyone else present besides the participants and researchers?	Methods > Data Collection, paragraph 2.
16. Description of sample	What are the important characteristics of the sample? e.g., demographic data, date	Results, opening paragraph.
**Data collection**		
17. Interview guide	Were questions, prompts, and guides provided by the authors? Was it pilot tested?	Methods > Focus Group Discussion Guide, paragraph 1; Supplementary Table 1. Pilot testing is addressed in Limitations, paragraph 1.
18. Repeat interviews	Were repeat interviews carried out? If yes, how many?	Not reported; appears not applicable because each participant took part in one FGD.
19. Audio/visual recording	Did the research use audio or visual recording to collect the data?	Methods > Data Collection, paragraph 2; Methods > Data Analysis, paragraph 1.
20. Field notes	Were field notes made during and/or after the interview or focus group?	Methods > Data Collection, paragraph 2.
21. Duration	What was the duration of the interviews or focus group?	Methods > Data Collection, paragraph 2.
22. Data saturation	Was data saturation discussed?	Methods > Data Analysis, paragraph 2. Saturation was not used

Item No and topic	Guide questions/description	Reported in manuscript section / location
		prospectively as the recruitment stopping criterion.
23. Transcripts returned	Were transcripts returned to participants for comment and/or correction?	Methods > Data Analysis, paragraph 1.
**Domain 3: Analysis and findings**		
**Data analysis**		
24. Number of data coders	How many data coders coded the data?	Methods > Data Analysis, paragraph 1.
25. Description of the coding tree	Did the authors provide a description of the coding tree?	Methods > Data Analysis, paragraph 1 (Codebook development and refinement).
26. Derivation of themes	Were themes identified in advance or derived from the data?	Methods > Data Analysis, paragraph 1.
27. Software	What software, if applicable, was used to manage the data?	Not reported; we used excel
28. Participant checking	Did participants provide feedback on the findings?	Methods > Data Analysis, paragraph 2.
**Reporting**		
29. Quotations presented	Were participant quotations presented to illustrate the themes/findings? Was each quotation identified? e.g., participant number	Results > all three theme sections; representative quotations are labelled with participant/FGD identifiers. Additional quotations are in Supplementary Table 2.
30. Data and findings consistent	Was there consistency between the data presented and the findings?	Results > all three theme sections.
31. Clarity of major themes	Were major themes clearly presented in the findings?	Results, overview paragraph; Results > Theme 1, Theme 2, and Theme 3 headings.
32. Clarity of minor themes	Is there a description of diverse cases or a discussion of minor themes?	Results, overview paragraph; Results > subtheme headings under Themes 1 to 3. Divergent/minor accounts are also described under Data Analysis.
