# Supplementary material for: Seeing the human behind the sample: How compassion training shaped inner awareness, relationships, and workplace meaning in HIV end-of-life research
Source: Palliat Care Soc Pract. 2026 Jul 8;20:26323524261467424. doi: 10.1177/26323524261467424 (PMC13351235; doi:10.1177/26323524261467424)
Supplement: Supplemental material - Seeing the human behind the sample: How compassion training shaped inner awareness, relationships, and workplace meaning in HIV end-of-life research [file sj-pdf-3-pcr-10.1177_26323524261467424.pdf]

Supplementary Table 3: Outstanding Quotes from the Last Gift Compassion Training Program Focus Group Discussion Participants

SUBTHEMES	PARTICIPANT IDs	QUOTATIONS
**THEME 1: INTRAPERSONAL GROWTH AND INNER AWARENESS**		
**Cultivating Mindfulness**	LG-CT-FGD-09	*I've had multiple attempts at creating kind of a regular meditation and mindfulness practice… having the accountability buddy was very helpful… I did have more trouble when I wasn't in my regular routine… traveling and things like that, missed a little bit more and had a little bit more trouble, but tried to kind of shift it to being in the airport and while waiting, doing a little bit of mindfulness instead of true meditation practice… since completing the study, so I did have a great run of about … 68 days of doing… 15 minutes of meditation every day after completing the study, which was great… even though the training was only four weeks, just having that and having the accountability buddies and seeing the benefits, you know, in real time, even though they're not instant… was helpful in reinforcing, but it really is important for me to have a daily practice, and then for bringing mindfulness to my research activities, … I've done more with bringing kind of compassion to the research activities.*
	LG-CT-FGD-15	*It's not that it isn't important, or that it isn't impactful. That isn't the challenging part. It's really like, in a busy day, to say, I'm going to make 10 minutes for myself, or I'm going to make five minutes for myself, and getting creative to do that in a way that can work in my life. And one of the things I do is… I just lay there in my car… And I will do my meditation before going into work, some days. So finding creative ways that work for me.*
	LG-CT-FGD-15	*But I do think in the day-to-day work and the day-to-day research, one thing I have benefited is if you can just take a step back… I've always known this. It's just I didn't have the discipline to do this or to incorporate this… and to take a step back and have that mindful awareness, and then go back into it and find that, oh, actually, there's an easier option, that I don't have to torture myself or my research colleagues…. And so this mindful awareness of sprinkling it throughout the day, I think I've noticed little nuggets of, "Oh, wait a minute. Why? Why am I so holding on strong? Do I have to do this this way?" So that's what I think it's helped me with.*
	LG-CT-FGD-16	*I need to find a way for myself where I can be more flexible like [LG-CT-FGD-15] is,…, rather than saying I have to be on my cushion at this time, and that's the only time I can do it…, if we have 10 minutes before I go jump to my next call, I will head upstairs, I'm working from home today, and do a quick 10 minute meditation, because even that reset makes a difference to me. And what I found is that I am able to find moments to pause where I'm washing dishes and I like, am not doing a podcast. I'm, you know, doing mindful washing of dishes, feeling the water, feeling things where it's, okay, I gave myself a moment…*
	LG-CT-FGD-25	*I was able to make it become more of a habit, like you almost have to treat it like brushing your teeth. You have to build it into your daily routine. So initially, I think there's challenges of trying to find time to commit, but I think once I created habits around it, it became easier.*
**Cultivating Self-Compassion**	LG-CT-FGD-02	*And there was a day where I called out because I just wanted to stay home… And I think when I confessed that it was just kind of like a big relief in the group, because… I kind of attended to myself first and… recognized that I needed a break, and so I gave myself that.*
	LG-CT-FGD-09	*I tend to minimize successes or accomplishments and just take them as givens and focus on the negatives. And so I feel like after the training and doing it, I have been able to consciously say, "No, this was an accomplishment"... and celebrate that a little bit and... take some pleasure and pat myself on the back a little bit more, which I think is helpful*
	LG-CT-FGD-15	*One of the exercises we did, is we had to write like, if a friend made a mistake, and how we would tell them, and then we had to write if I made the mistake, how I would talk to myself. And it was very apparent to me of how different that was, and how I was so harsh on myself that I've actually, because of that exercise, I started this practice where I wrote a letter, a compassionate letter, to myself,*
	LG-CT-FGD-16	*That is also self-compassion, and [when] it falls outside of those three buckets, then I can kindly say "No", and that's okay, and I can be okay with that, because in the past, I think, I'll be honest, and I would, you know, it also has impacted the way I've worked, like in the past, like I have colleagues that I'll be working, and I can see emails coming through at like, 8, 8 p.m. when I'm still doing stuff with my kids. In the past, I'll be like, "oh, you should be working" and talking negatively to myself, been like, maybe you should be "Go, go, do that instead". And I can say to myself, "Okay, that person is in a different life stage than me. Their kid is in bed. It's okay for me to take time for myself right now with my children*

		and work on this at a time that works for me", so moved from… more internal self-flagellation to more self-compassion.
**Becoming More Open to Accepting/ Responding to Compassion**	LG-CT-FGD-02	*I think with strangers like I used to love not being perceived in public... now, after the compassion training, I find myself like smiling to strangers... And I think it's something so small, but it makes a difference, at least in my day when like a stranger smiles at me.*
	LG-CT-FGD-03	*It's hard, because I always grew up with this thing, thinking that… if people is compassion with you, it's because they want something or they need something, but it's not always true. So nowadays I do accept it and try to let go of my fear of, "oh, they're being nice because they want something.*
	LG-CT-FGD-06	*When people are being compassionate towards me, it always throws me off, in a way, because I'm not really sure what to do with it... when we talk about being compassionate to yourself, it definitely helps just being like this is the normal thing, and this is a positive thing. It's not like some very rare thing that happens.*
	LG-CT-FGD-07	*…just kind of like random acts of kindness… I was running, and these ladies, like, they said, "Good job."… And I was just like, "Oh my gosh. Like this is such an infectious, like, energy, right?" And so the next lady that I passed, I said, "Oh my god, good job." … I just feel like it's trying to carry the energy of… happiness… a little brief moment where you're like, "wow, that stranger was so kind to me". Like, they don't know anything about me, but they, like, did this for me, and it just means so much more when, like, a stranger does it because… they don't have any ties to you. There's no advantage, right? They don't have any conflict of interest. And so it really shows kind of like that genuine connection that you… truly just want to do good towards anybody, not just because you have, like, a certain interest in getting an advantage or something.*
		Before the training… I felt like when other people expressed compassion towards me, I didn't want to like burden them. Like I thought that them being compassionate towards me would be like a burden that would put on them to kind of like, look out for me, I guess, or help me or something. And I think after the training… I'm more kind of receptive towards compassion, just because if I am willing to be compassionate for somebody else, I'd want somebody to accept my compassion, so I kind of try to view it in that lens.
	LG-CT-FGD-16	*So I would say In the past, like even being the center of attention in any way, like I felt very awkward, and I still do, but I will say that has dropped. That discomfort with compassion or being highlighted has improved in that I can respond to compassion from other people better, slightly.*
**THEME 2: INTERPERSONAL AND COMMUNITY CONNECTIONS**		
**Recognizing Common Humanity & Extending Compassion to Others**	LG-CT-FGD-02	*I think it's made me more comfortable being uncomfortable, like living in the discomfort, because sometimes being compassionate can be really uncomfortable… it's uncomfortable being compassionate sometimes, because it's like… exiting your ego, I guess, and you're trying to enter this like new like view of seeing their perspective. And, yeah, I think it's just letting go of your ego and just, you know, giving each other like the best.*
	LG-CT-FGD-03	*Because of this training… personally I started… going to people and say, "Do you need help? Do you need this? Are you okay? Or do you need to go?" You know, stuff like that. So more conscience of other people's tiredness, or maybe they have issues, you know, before they left home or whatever.*
	LG-CT-FGD-09	*I think through the training, I had a change in perspective where those people may appear to have, you know, everything together, but their challenges are just different challenges than mine. So while those challenges aren't outwardly on display. You know, they have the same shared experience, per se, with a challenge, it just may not be the one that I have… while theirs is not externalized, they, they still have their own challenges that are unique to them. But… each of us has the challenges that we have. And each of us struggled in our own way, but the commonality is that we have struggles.*
	LG-CT-FGD-15	*I think it starts like when you can have compassion for yourself, then it's a trickle-down effect where I can have better compassion throughout. If I can be compassionate to myself, then I can also think this person's going through a hard time. So can I say this in a kinder way?... So it's almost like this has given me clarity to have compassion for myself, but also an empowerment to have compassion for myself and for others… I think the difficult person, I think it really stems to the shared human experience that we all want similar things, right? Peace, comfort, love, all of that. I do better some days than others on that for, you know, extending that to myself and others… I think that would be wonderful, is to strengthen that muscle of compassion for ourselves and compassion for others in the research and in the work and in the life that we lead.*
**Navigating Interpersonal**	LG-CT-FGD-01	*For me, one of the things, other than not taking anything personal is like, I kind of used to be… I just had to be right. And I realized that, "yes, I can be right for the rest of my life, but I'll probably lose a lot of important people and what is important… to me." And so once I*

**Conflict**		*was okay with not being right, which for me, also means not having an ego,… in interpersonal relationships, not needing to be right has prevented a lot of unnecessary conflict… this training reinforced that.*
	LG-CT-FGD-06	*I've had a couple of difficult people that I've talked to since the compassion training, and I think the biggest thing for me was just patience. I think that really helped me not sort of continue to… make it more difficult of an interaction… being more compassionate towards this person and not fighting with them, necessarily. Because I tend to try to run the exact opposite of what someone's saying if I don't agree with them, just for the sake of it, and that's not helpful… definitely, that's helped me sort of be in a better mindset towards talking to some difficult people, if I have to.*
	LG-CT-FGD-15	*The place that I think I've focused, consciously or unconsciously, was with difficult people… So really trying to consciously, when I do meditate, especially if I am struggling with them, or even if I haven't struggled them in a while. But I know it's almost like being ready the next time I interact with them… And I do feel like there's been small changes, and even I would say big changes, of finding space and compassion and… having a better understanding of, you know, that they also want peace and they're just, maybe they're struggling to find it.*
	LG-CT-FGD-18	*I was better in trying to understand the people I supervise here… I think I got better at that, like, try to be in their shoes and see if it's not all about work, right? So maybe they are struggling at work because there is something else outside work. Usually, that's the way it is, so I think I got better there.*
**THEME 3: APPLYING COMPASSION SKILLS TO WORK AND EVERYDAY LIFE**		
**Building Community in Workplace Culture**	LG-CT-FGD-06	*I think that for me, at least it just everything feels a lot more coherent. And I feel like we've only had one autopsy, I think, since the last training. So that one definitely was, I don't want to say a trial run, but it felt a little bit different. It felt more like people were checking in a little bit more than average, I would say. So I think like it as a group, it feels like we're more communicative in that aspect. Yeah, yeah, so far.*
		It just worked really well. We were all… checking in with each other, and yet we were still getting things done… The compassionate aspect of it really was positive, I think definitely, at least from what I saw
	LG-CT-FGD-07	*During the compassion training itself, I think we had exercises where we had to, like, meet up with one of our coworkers and be somewhat vulnerable… And so it really created that space. And so I think that we kind of opened up that dialogue, which made it easier to communicate, you know, even through the flow of, like, the autopsy, when it's happening even in the lab. And so you just kind of build… a relationship with your coworkers*
		In the last autopsy… it's been like, the most efficient. I think that it builds more comfortable and closer relationship with each of my coworkers. And so I'm able to ask them questions about, "What can I help them with more comfortably?" and without, like, this awkwardness? And so I think that barriers come down, and we're able to build closer relationships… it feels so beautiful to be a big part of, like, a bigger, represent… there's so many people involved in this.
	LG-CT-FGD-09	*So I think it does give an extra sense of community… within the team, just to have been able to share and recognize that we all have a lot of similar thoughts and inner dialogues… to be able to have that additional peer support and validation of feelings… but basically, you know, reinforcing that the compassion, as is a big component of the of the study itself, and that extends to the staff members.*
**Supporting Stress, Resilience, and Work Meaning**	LG-CT-FGD-02	*I think before this training, it was really easy to think of when I think of Last Gift, it was just like the six of us and like our small team. But I think after completing this training, it's a lot easier to see this as like a really big picture, where before it was really easy to say, like, "oh yeah, I work in an HIV lab," but now it's like, "oh, I work in an HIV lab that does Last Gift"…Like, there's so much more than just, like, our little autopsy team. There's like, I'm sure there's more people we don't even know. Like, there's just so many people behind this that make the picture so much bigger, and it feels really nice to be part of something like this, and I think it's very unique, and I think it's something that we can all talk about in like decades from now.*
		And I think the stress, like, just in myself, I think it decreased it, because it's like, I'm recognizing when I need more compassion… compassion can be a verb, and it's just kind of like acting upon it and giving myself that. And stress associated with Last Gift study, I think it's a good stress, because you want to honor the person… So I think it's that kind of stress where, like you want to complete their last wish.
	LG-CT-FGD-03	*It's less stress than before. So it did, the compassion training, it's it keeps helping me a lot, knowing that there's people around me that I can talk to, or coworkers that, instead of coworkers became friends, and they're there for me.*

	LG-CT-FGD-06	*The biggest part of me, before this compassion training, is… you would have a tube, and you wouldn't think of it as a piece of person. It was just the tube… stepping in that compassionate mindset… has definitely made it feel more worthwhile. Not that it didn't initially… but it felt more of a personable factor... definitely being more mindful of that and stuff, definitely just reminds me that… we're not just doing stuff just for the sake of seeing numbers. There's an outcome that's that we're going for, and people are donating a lot of themselves to figuring out what these numbers are.*
	LG-CT-FGD-09	*The fact that this was afforded to people you know within the Last Gift team and beyond, I think, went a long way to show the priorities of the Last Gift team and leadership and investigators and the team as a whole, to actually to emphasize this and focus on making sure that we're taking care of ourselves. And so I think just having the training and going through, you know, all of this, in the investment in allowing everybody to have the time to do this, meant a lot.*
		I think that was helpful seeing that all of the different people and different aspects of people associated with the Last Gift come together for this study.
		It helped me to step back and kind of reflect on that and remember to think about the next of kin and to think about the even the people standing next to me at the autopsy table, and kind of extend some of that compassion and a little bit of grace for myself and for everyone else on the team, and then the participants and their next of kin. And just to also helped me, instead of necessarily pushing away kind of negative feelings or sad feelings,.. , allow myself to feel them and kind of work through them, instead of just trying to feel like I have to be super professional all the time and suppress those feelings as they come up… I think I had drifted a little bit from the participant center aspect of it. And so this was good to reconnect with that and help re-motivate me on the importance.
	LG-CT-FGD-15	*I was going through a stressful time, and it helped me… have better perspective, mindful awareness, the meditation, even the small group discussions, really were able to help me process… the stress… And so, I definitely think it did help me with coping with my stress. And it wasn't like, I was hearing other people's stress, and I didn't feel like I was carrying other people's stress, so if that helps.*
	LG-CT-FGD-16	*I realized how much I really actually needed it… If anything, it decreased my emotional distress in the way that it helped me slow down in many pieces of my life… I think I'm slowly but surely able to compartmentalize a little bit more so that,… not in a bad way, but in a way where I don't bring home as much of the emotional toll that the patient care is taking on me.*
